# Pioglitazone prevents cholesterol gallstone formation through the regulation of cholesterol homeostasis in guinea pigs with a lithogenic diet

**DOI:** 10.1186/s12944-019-1159-4

**Published:** 2019-12-11

**Authors:** Tao Han, Yangge Lv, Shijia Wang, Tao Hu, Hao Hong, Zan Fu

**Affiliations:** 1 Department of Intensive Care Unit, the First Affiliated Hospital of Nanjing Medical University, Nanjing, 210000 Jiangsu China; 2 Department of Pharmacology, China Pharmaceutical University, Nanjing, 210009 China; 3 Department of General Surgery, the First Affiliated Hospital of Nanjing Medical University, Nanjing, 210000 Jiangsu China

**Keywords:** Pioglitazone, Gallstone, Lithogenic diet, Cholesterol, Bile acid

## Abstract

**Background:**

The cholesterol gallstones diseases (CGD) is highly correlated with metabolic syndrome and type 2 diabetes. The present study aimed to investigate preventive effects of pioglitazone (PIO), an antidiabetic drug, on the CGD in guinea pigs fed with a lithogenic diet (LD).

**Methods:**

The guinea pigs were fed with the LD for 8 weeks. All guinea pigs were grouped as follows: low fat diet; LD; LD plus PIO (4 mg/kg); LD plus PIO (8 mg/kg); LD plus ezetimibe (EZE) (2 mg/kg). Gallbladder stones were observed using microscopy. The profile of biliary composition, and blood glucose, insulin and lipid were analyzed. The liver or ileum was harvested for determinations of hydroxyl-methyl-glutaryl-CoA reductase (HMGCR), sterol regulatory element-binding proteins 2 (SREBP2), 7α-hydroxylase (CYP7A1), adenosine triphosphate-binding cassette (ABC) sterol transporters G5 and G8 (ABCG5, ABCG8), bile salt export pump (BSEP), Niemann-Pick C1-Like 1 (NPC1L1) and acetyl-coenzyme A cholesterol acyltransferase (ACAT2) by Western blot. The gallbladders were used for histological examination.

**Results:**

The LD successfully induced gallstone. Both pioglitazone and ezetimibe prevented gallstone formation, as well as hepatic and cholecystic damages. Pioglitazone significantly decreased HMGCR and SREBP2, but increased CYP7A1, ABCG5, ABCG8, and BSEP in the liver. Pioglitazone also remarkably decreased NPC1L1 and ACAT2, while increased ABCG5/8 in the intestine. The beneficial alterations of cholesterol and bile acids in the bile, as well as profile of glucose, insulin and lipid in the blood were found in the guinea pigs treated with pioglitazone.

**Conclusion:**

Pioglitazone has a noticeable benefit towards the CGD, which is involved in changes of synthesis, transformation, absorption, and transportation of cholesterol.

## Introduction

Cholesterol gallstone disease (CGD) is one of the most prevalent and costly diseases. Aging, high calorie diet, hyperinsulinism and metabolic syndrome are among the preeminent risk factors for developing CGD [1]. Major pathogenetic mechanisms are involved in hepatic hypersecretion of cholesterol [2], gallbladder motility disturbances [3], gallbladder inflammation [4], and increased intestinal absorption of cholesterol [2], and altered gut microbiota [5]. Precipitation of excess cholesterol in bile as solid crystals is a prerequisite for cholesterol gallstone formation. Novel approaches against these pathogenetic factors are urgently needed to be developed for the prevention and treatment of the CGD.

Dietary cholesterol is trafficked into enterocyte by Niemann-Pick C1-like 1 (NPC1L1) protein [6], and can be reversely transported to the intestinal lumen by adenosine triphosphate-binding cassette (ABC) sterol transporters G5 and G8 (ABCG5, ABCG8) [7, 8]. After cholesterol passes through the enterocyte membrane, it is esterified by acetyl-coenzyme A cholesterol acyltransferase 2 (ACAT2), and subsequently transported in the chylomicrons circulating then reaches the liver for biliary secretion [9, 10]. HMG-CoA reductase (HMGCR) and 7α-hydroxylase (CYP7A1) play important roles in maintaining cholesterol homeostasis. HMGCR is a rate-limiting enzyme in hepatic cholesterol biosynthesis while CYP7A1 is a rate-limiting enzyme transforming cholesterol to bile acid (BA). The ABCG5/8 effluxes sterols including cholesterol from hepatocytes and enterocytes into the bile and intestine, respectively, and may therefore simultaneously increase biliary cholesterol excretion and lower plasma cholesterol levels [8]. The bile salt export pump (BSEP), also known as ABCB11, is the major canalicular BA transporter [11]. Cholesterol gallstone formation is associated with downregulation of BSEP expression on the canalicular membrane of hepatocytes [12]. In addition, cholesterol metabolism is tightly controlled by some feedback mechanisms, including regulation of the sterol regulatory element-binding protein 2 (SREBP2) and liver X receptor α (LXRα) [13–15].

Pioglitazone, a thiazoledinedione (TZD), is a highly selective peroxisome proliferator-activated receptor-γ (PPARγ) agonist. Widely used as antidiabetic agent, pioglitazone not only has an impact on glycemic control but also has been shown to increase HDL and decrease LDL and TG in diabetes [16]. All anti-diabetic drugs have varying effects on lipid profile but overall pioglitazone has shown more favorable lipid lowering effect compared to other antidiabetics including rosiglitazone [17, 18]. In more recent years, the focus on pioglitazone has been intensified due to its novel biological roles. Herein, we investigated effects of pioglitazone on cholesterol gallstone formation as well as NPC1L1, ACAT2, HMGCR, CYP7A1, ABCG5/8, and BSEP in the guinea pigs with a lithogenic diet.

## Materials and methods

### Materials

Pioglitazone (PIO) was purchased from MSD Pharma (Singapore) Pte. Ltd. Ezetimibe (EZE) was purchased from DEYUAN PHARM Co. Ltd. (Shanghai, China). The antibodies of SREBP-2(sc-13,552), CYP7A1 (sc-293,193), FXR (sc-25,309), LXR (sc-271,064), BSEP (sc-74,500), CCK-AR(sc-514,303) and NPC1L1(sc-166,802) were purchased from Santa Cruz Biotechnology Co. Ltd. (Santa Cruz, CA). The antibodies of HMGCR (ab174830), ABCG5 (ab87116) and ABCG8 (ab126493) were from Abcam Technology Co. Ltd. (Cambridge, UK). The antibodies of ACAT2 (13294S) and PPARγ (2443S) were from Cell Signaling Technology Inc. (Boston, USA). Histone H3, β-actin and the secondary antibodies were from Bioworld Technology Co. Ltd. (Minneapolis, MN, USA). BCA assay kit was from Beyotime (Nanjing China). Amplex Red cholesterol (CH) assay kit was from the Invitrogen (USA). All other chemical or Biological reagents were commercially available.

### Animals

Male guinea pigs (350 ± 20 g) were obtained from the Yangzhou University Medical Center (Yangzhou, China) and were housed under the environmentally controlled conditions of 12-h light-dark cycles in a full-barrier facility with a relative humidity of 60 ± 5% and temperature of 22 ± 2 °C. The guinea pigs had free access to water and food. All diets were prepared and pelleted by Yangzhou University Medical Center. The lithogenic diet (LD) refers to chow diet supplemented with approximately 1.25% cholesterol, 0.5% bile salt and 15% fat as well as 0.0033% or 0.0067% pioglitazone or 0.0017% ezetimibe. After 1 week of adaptation, the guinea pigs were randomly divided into 5 groups and treated as follows: low fat diet (CTRL), LD, LD plus PIO 4 mg/kg (PIO-L), LD plus PIO 8 mg/kg (PIO-H), LD plus EZE 2 mg/kg. The guinea pigs were fed with the LD containing pioglitazone or ezetimibe for 8 weeks. The experiments were performed according to the National Institutes of Health Guide for the Care and Use of Laboratory Animals (NIH publication No. 86–23, revised 1996), and approved by the guidelines of the Institutional Animal Care and Use Committee of China Pharmaceutical University.

### Biochemical assays

The levels of blood glucose, triglyceride (TG), low-density lipoprotein CH (LDL-C) and high-density lipoprotein CH (HDL-C) were measured according to the instructions of their assay kits (Nanjing Jiancheng Bioengineering Institute, Nanjing, China). The serum insulin was measured using the insulin ELISA kit (Millipore, USA). The CH, BA, phospholipid (PL) in the serum or bile were measured using the respective kits. Furthermore, the CH saturation index (CSI) was calculated using the following equation: actual molar percentage of CH in bile/the largest soluble CH concentration at a given bile molarity in the Carey table [19].

### Histological evaluation of the liver and gallbladder

The liver and gallbladder specimens were prepared and fixed in 4% neutral-buffered formaldehyde overnight. The specimens were embedded in paraffin, cut into 4-μm-thick slices, stained with haematoxylin and eosin (H & E). After dewaxing, staining, dehydration, transparency and sealing, the specimens of liver and gallbladder were examined under a light microscope.

### Western blot analysis

Liver and ileum were chopped into small pieces and homogenized in 0.5 ml of lysis buffer (phenylmethylsulfonyl fluoride (PMSF): RIPA = 1:9). The dissolved proteins were collected from the supernatant after centrifugation at 12,000×g for 15 min. Protein concentration was determined by BCA assay kit (Beyotime, Nanjing China). The protein was separated on 12% SDS-polyacrylamide gel and transferred onto the PVDF membrane. After blocking the nonspecific site with blocking solution (5% nonfat dry milk), the membrane was incubated overnight with specific primary antibody at 4 C with primary antibodies: HMGCR (1:300), SREBP-2 (1:500), CYP7A1 (1:500), ABCG5 (1:600), ABCG8 (1:300), LXRα (1:500), BSEP(1:500), NPC1L1(1:500) and ACAT2(1:1000). Histone H3 (1:1000) and β-actin (1:3000) served as an internal control, respectively. Subsequently, the membrane was washed with TBST followed by incubation with appropriate horseradish peroxidase-conjugated secondary antibody (1:5000) at the room temperature for 1 h. The blots were again washed with TBST and then developed with the ECL Plus Western Blotting Detection System (Tanon Science & Technology Co. Ltd.)

### Statistical analyses

The data were expressed as means ± SD All data, unless specified, were analyzed by a one-way ANOVA followed by a Dunnett’s post hoc analysis for multiple comparisons. All analyses were carried out using SPSS, version 20.0. A probability value (*P*) of <0.05 was considered statistically significant.

## Results

### Pioglitazone treatment inhibits cholesterol gallstone formation in the Guinea pigs fed with LD

**Fig. 1:**
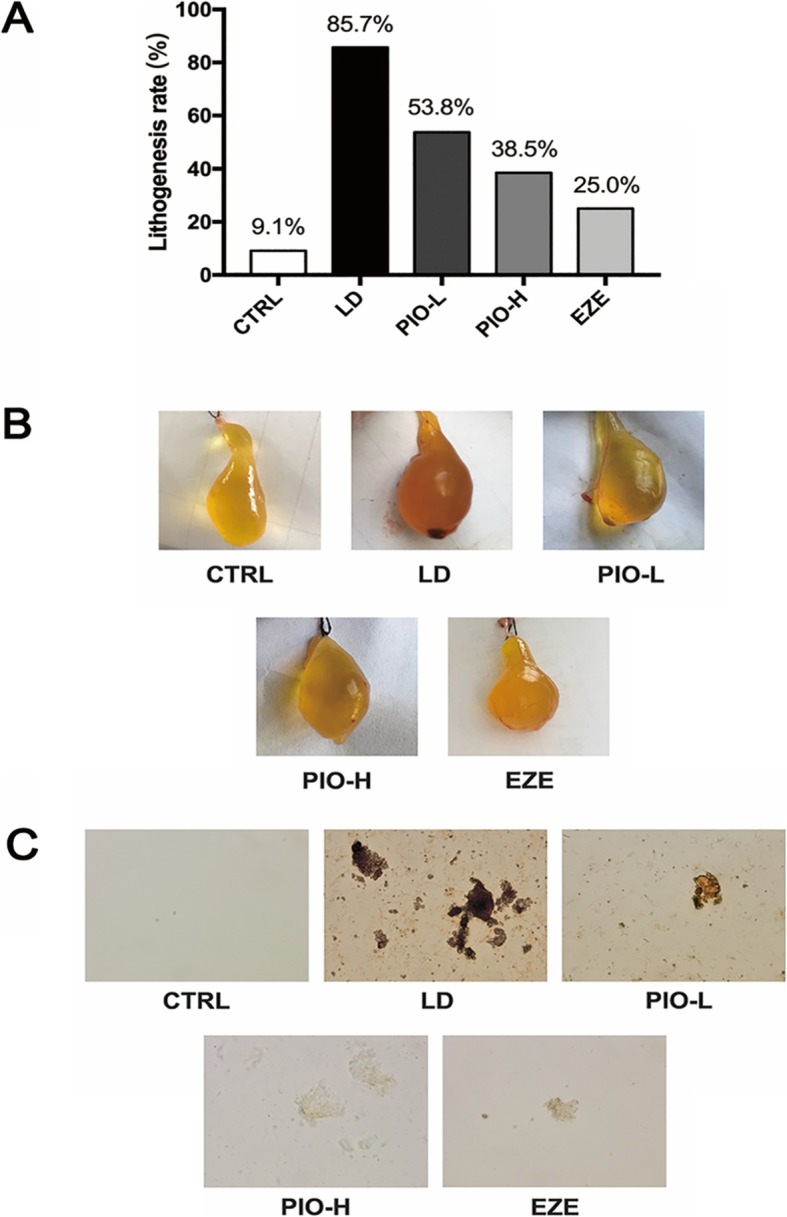
Pioglitazone treatment inhibits cholesterol gallstone formation in the guinea pigs fed with LD. **a** lithogensis rate; **b** macroscopic appearances of the gallbladders; **c** photomicrographs of bile, liquid crystals and gallstones

In our study, the CTRL group had a cholesterol gallstone formation rate of 9.1%, the LD group showed a cholesterol gallstone formation rate of 85.7%, a statistically significant increase over the rate of the CTRL group. The gallstone formation rate in the PIO-L and PIO-H groups were 53.8 and 38.5%, respectively. The gallstone formation rate was 25.0% in the guinea pigs treated with a hypolipidemic agent, ezetimibe, as positive control. Thus, both of pioglitazone and ezetimibe reduced the rates of gallstone formation in the guinea pigs. The Fig. 1c shows photomicrographs of the bile, liquid crystals, and gallstone samples. The bile of control guinea pigs did not contain any crystals or sludge, while the bile of the LD-fed guinea pigs contained sludge particles and crystals. The PIO-L, PIO-H or EZE group contained few crystals and sludge in their bile samples.

### Pioglitazone treatment alters biliary composition and CSI in the Guinea pigs fed with LD

As shown in Table 1, the levels of both CH and BA were significantly increased in gallbladder bile of the guinea pigs in the LD group (**P < 0.01), compared with those in the CTRL group. The CH level significantly declined and BA level further increased in the gallbladder bile after treatment with high-dose pioglitazone or ezetimibe (**P < 0.01). Treatment with low-dose pioglitazone also changed levels of biliary CH and BA, but there were not statistical significances. In addition, treatment with low- or high-dose pioglitazone and ezetimibe significantly inhibited the LD-induced increase in CSI in the guinea pigs.

**Table 1:** Pioglitazone treatment alters biliary composition and CSI in the guinea pigs fed with LD

Variables	CTRL	LD	PIO-L	PIO-H	EZE
CH (mmol/L)	2.93 ± 1.14^**^	8.39 ± 1.64	7.05 ± 1.68	5.65 ± 1.33^**^	5.07 ± 1.30^**^
BA (mmol/L)	56.09 ± 9.86^**^	77.02 ± 9.86	80.59 ± 9.77	92.26 ± 8.46^**^	85.47 ± 8.91
PL (mmol/L)	22.89 ± 5.15	25.65 ± 4.89	26.33 ± 4.68	27.47 ± 5.27	26.22 ± 4.56
CSI	0.57 ± 0.17^**^	1.13 ± 0.15	0.85 ± 0.13^*^	0.70 ± 0.13^**^	0.67 ± 0.10^**^

Values are expressed as means ± SD (*n* = 8). ^∗^*P < 0.05* and ^∗∗^*P < 0.01* versus LD group

### Effects of pioglitazone on metabolic parameters

As shown in Table 2, compared with CTRL group, treatment with the LD had no significant effects on blood glucose and insulin, while treatment with high-dose pioglitazone significantly decreased levels of blood glucose and insulin (blood glucose: **P < 0.01; insulin: *P < 0.05). The levels of CH, LDL-C and TG increased and HDL-C level decreased in the peripheral blood of the guinea pigs after feeding LD for 8 weeks (**P < 0.01). Treatment with high-dose pioglitazone or ezetimibe significantly decreased CH, LDL-C and TG levels, and increased HDL-C level in the serum of guinea pigs (CH: **P < 0.01; LDL-C: *P < 0.05; TG: **P < 0.01; HDL-C: *P < 0.05). Treatment with low-dose pioglitazone showed some tendency of regulation in blood glucose, insulin and lipid profile, but did not reach statistical significance (*P > 0.05*).

**Table 2:** Effects of pioglitazone on metabolic parameters

Variables	CTRL	LD	PIO-L	PIO-H	EZE
Body weight (g)	479.39 ± 50.97	511.83 ± 70.00	490.13 ± 40.78	435.46 ± 69.62^*^	492.46 ± 63.29
Blood glucose (mmol/L)	3.35 ± 0.52	3.90 ± 0.63	3.39 ± 0.68	2.99 ± 0.39^**^	3.43 ± 0.51
Insulin (mmol/L)	2.45 ± 0.53	3.00 ± 0.54	2.58 ± 0.59	2.40 ± 0.53^*^	2.51 ± 0.29
CH (mmol/L)	3.60 ± 0.74^**^	6.28 ± 0.91	5.85 ± 0.77	5.04 ± 1.07^*^	4.36 ± 0.81^**^
TG (mmol/L)	0.37 ± 0.07^**^	0.63 ± 0.10	0.50 ± 0.10^*^	0.44 ± 0.10^**^	0.47 ± 0.09^**^
LDL-C(mmol/L)	1.39 ± 0.19^**^	2.02 ± 0.30	1.84 ± 0.27	1.72 ± 0.27^*^	1.64 ± 0.18^*^
HDL-C (mmol/L)	1.03 ± 0.20^**^	0.69 ± 0.19	0.77 ± 0.14	0.91 ± 0.10^*^	0.94 ± 0.18^*^

Values are expressed as means ± SD (n = 8). ^∗^*P < 0.05* and ^∗∗^*P < 0.01* versus LD

### Pioglitazone treatment prevents the histological damages of liver or gallbladders in the Guinea pigs fed with LD

**Fig. 2:**
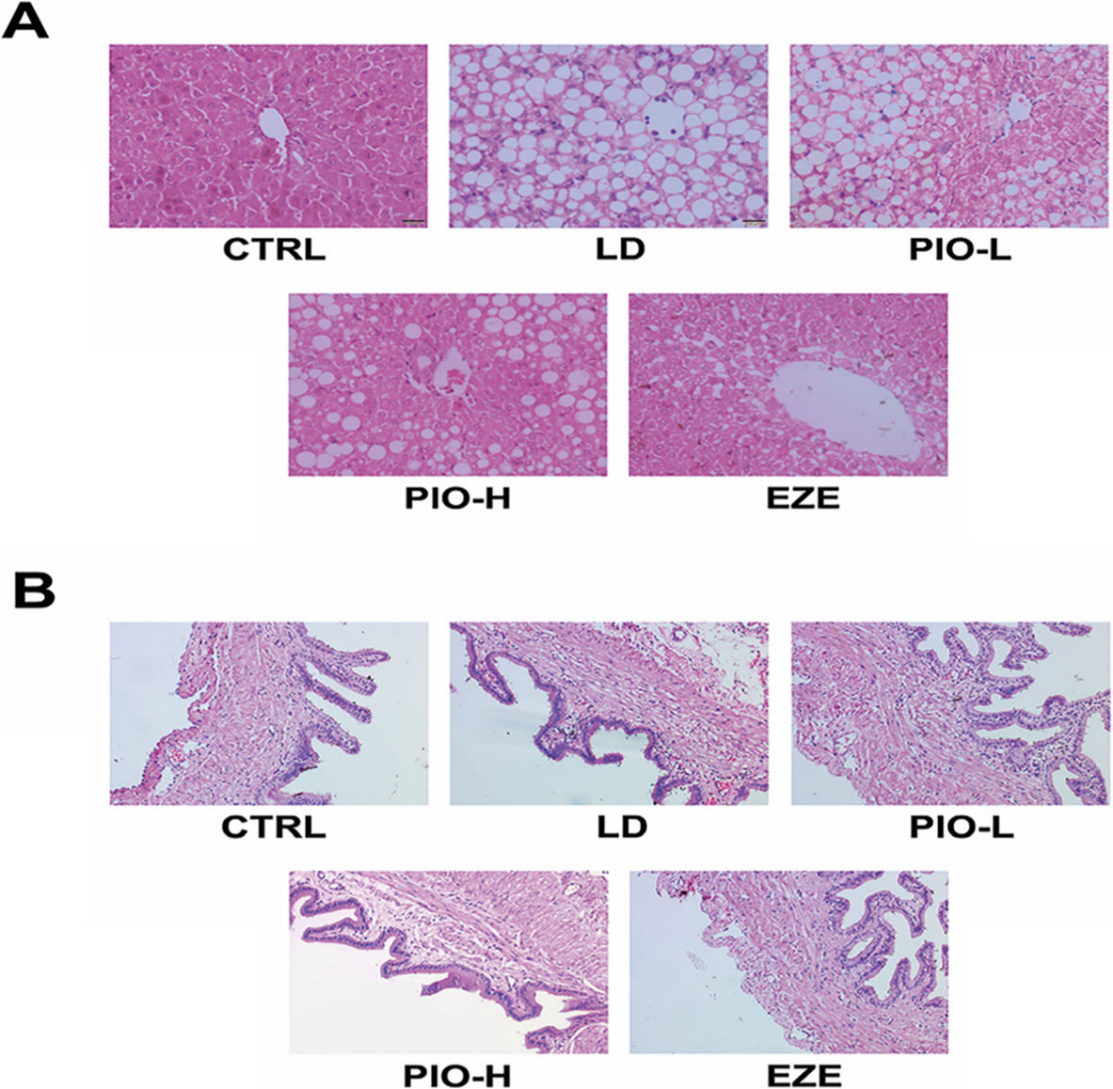
Pioglitazone treatment prevents the histological damages of liver or gallbladder in the guinea pigs fed with LD. **a** Histological examination of the liver by H&E staining. **b** Histological examination of the gallbladder by H&E stainin

The structure of the normal liver was as follows: hepatic plates radiate from the central vein, and the plates are separated at irregular intervals by sinusoids without abnormal morphological changes. Some characteristic histology features of the CGD were observed in the livers of the guinea pigs fed with LD for 8 weeks, including steatosis, spotty necrosis, hepatocyte ballooning, and lobular inflammation. Compared with the LD group, the extents of hepatocellular steatosis, necrosis and hepatocyte ballooning were markedly decreased in the PIO-H and EZE groups. These results indicated that both ezetimibe and pioglitazone treatment produced protective effects on the liver damage induced by the LD (Fig. 2a). Gallbladder inflammation is indicated by thickened gallbladder wall, infiltrated inflammatory cells in the stromal layer, and submucosal vasodilatation in the CGD. All these signs were seen in the LD group, but few inflammatory signs were observed in the PIO-H and EZE groups (Fig. 2b).

### Pioglitazone promoted LD-induced decrease in HMGCR or SREBP-2 and inhibited the decrease in CYP7A1 in the liver

**Fig. 3:**
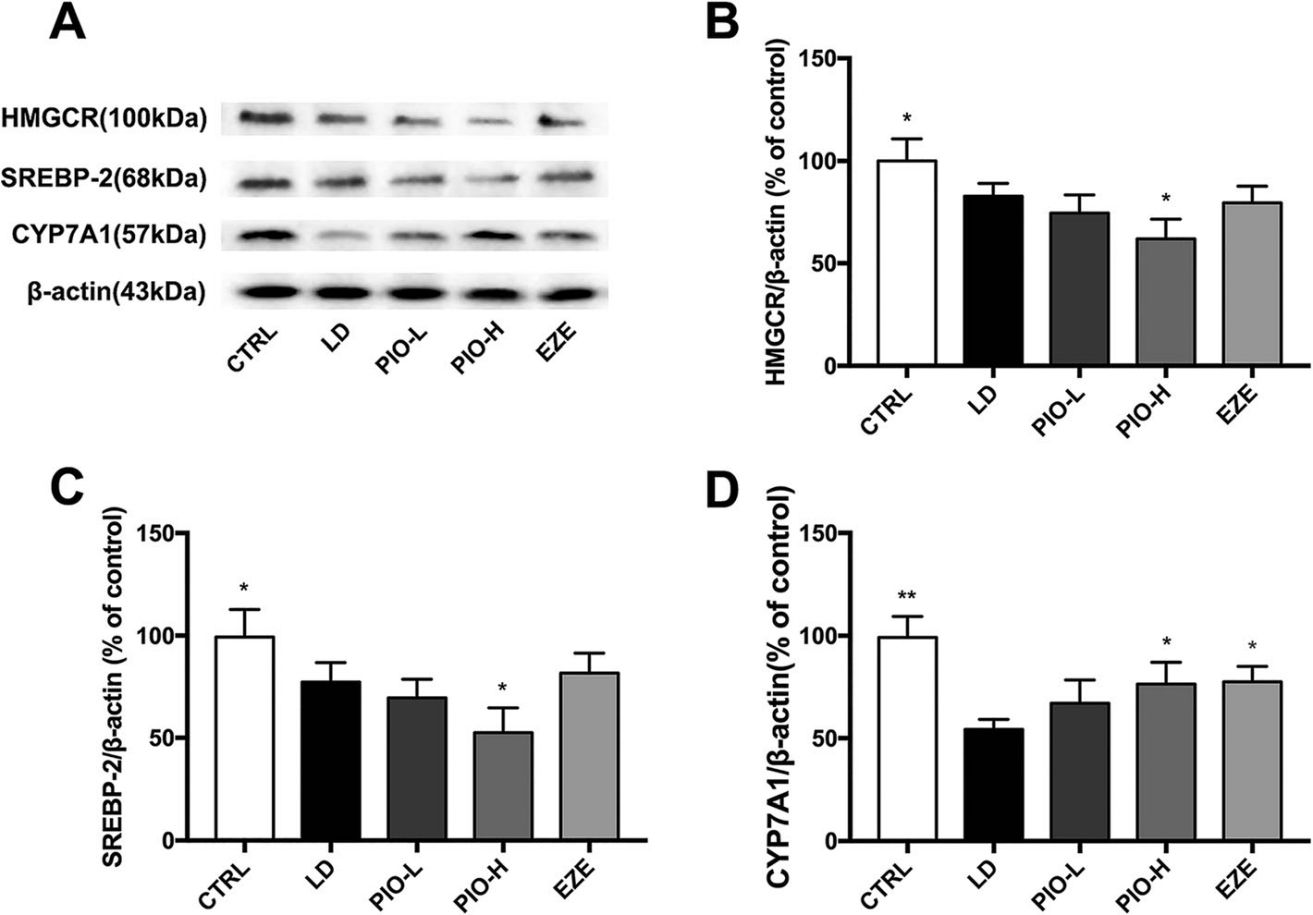
Pioglitazone promotes LD-induced decrease of HMGCR or SREBP-2 and inhibited decrease in CYP7A1 in liver. **a** Western blot analysis of HMGCR, SREBP-2 and CYP7A1 protein; **b** Quantification of HMGCR expressed as the ratio (as a percentage) of control; **c** Quantification of SREBP-2 expressed as the ratio (as a percentage) of control; **d** Quantification of CYP7A1 expressed as the ratio (as a percentage) of control. Values shown are means ± SD, *n* = 4. *p < 0.05 and **p < 0.01 versus LD group

Western blot was employed to examine the expression of HMGCR or SREBP-2 in the liver of guinea pigs challenged with LD plus different drugs or the LD alone for 8 weeks. The Fig. 3 showed that LD caused significant decreases in HMGCR and SREBP-2 (*P < 0.05), and high-dose pioglitazone enhanced the decreases in both proteins in the liver of guinea pigs (*P < 0.05). However, ezetimibe didn’t produce same effects. In addition, LD induced significant reduction of CYP7A1 expression in the liver (**P < 0.01), and this effect was reversed by pioglitazone (*P < 0.05). Ezetimibe also produced similar effect. These results show that pioglitazone like ezetimibe reduces biosynthesis and transformation of CH in liver.

### Pioglitazone increased the expression of hepatic ABCG5, ABCG8 and BSEP

**Fig. 4:**
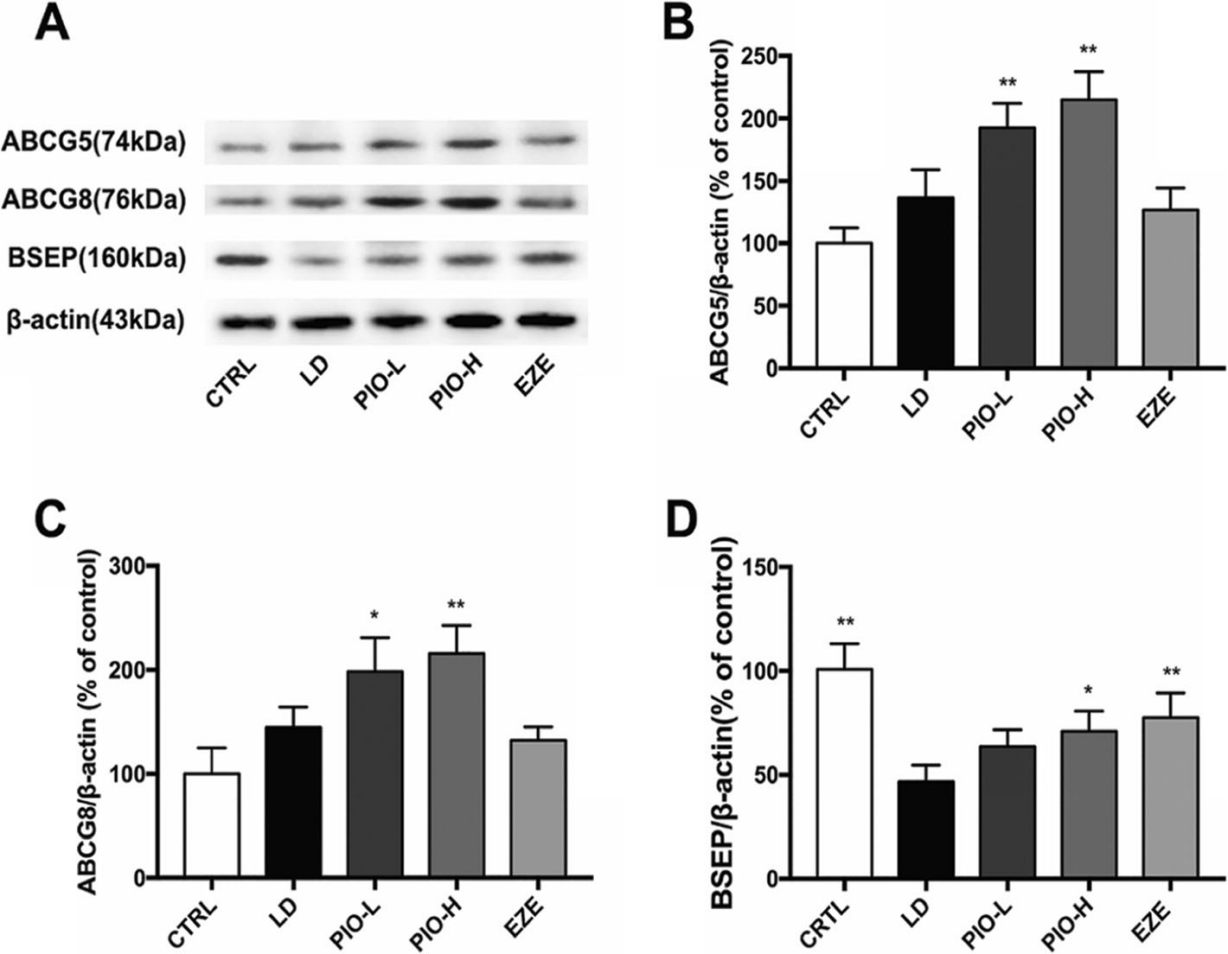
Pioglitazone increases expression of hepatic ABCG5, ABCG8 and BSEP. **a** Western blot analysis of ABCG5, ABCG8 and BSEP protein; **b** Quantification of ABCG5 expressed as the ratio (as a percentage) of control; **c** Quantification of ABCG8 expressed as the ratio (as a percentage) of control; **d** Quantification of BSEP expressed as the ratio (as a percentage) of control. Values shown are means ± SD, *n* = 4. *p < 0.05 and **p < 0.01 versus LD group

As shown in Fig.4, LD didn’t change the expression of ABCG5 and ABCG8, but LD plus low- high- dose pioglitazone significantly increased expression of ABCG5 and ABCG8 in the liver (ABCG5:**P < 0.01; ABCG8: *P < 0.05). Ezetimibe didn’t show these effects. Pioglitazone and ezetimibe remarkably inhibited the LD-induced decrease of BSEP in liver (BSEP: *P < 0.05, **P < 0.01). These results suggest that pioglitazone could increase the transport of CH or BA in liver.

### Pioglitazone increased ABCG5 and ABCG8 and decreased NPC1L1 and ACAT2 in the intestine

**Fig. 5:**
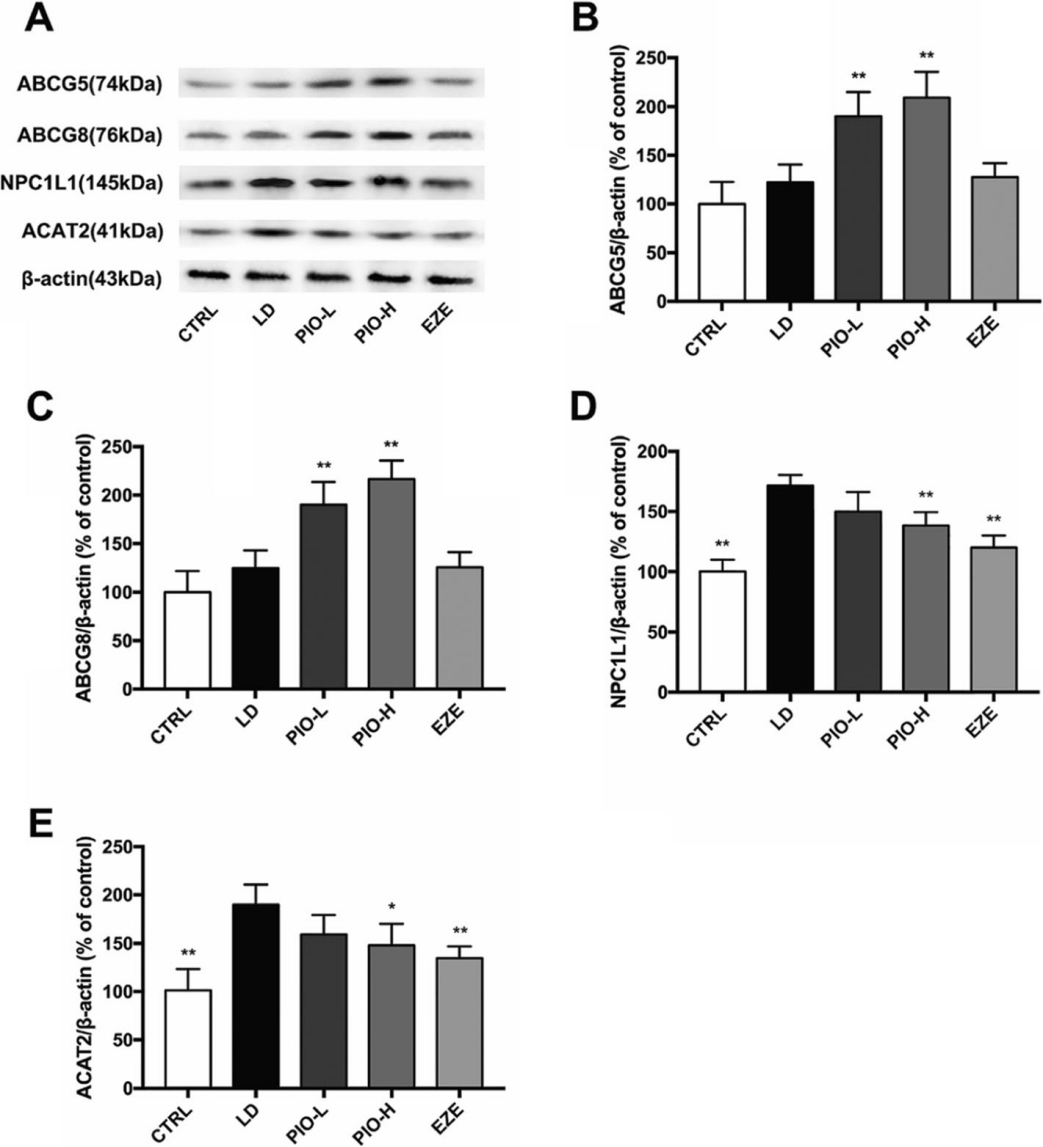
Pioglitazone increases ABCG5 and ABCG8 and decreases NPC1L1 and ACAT2 in intestine. **a** Western blot analysis of ABCG5, ABCG8, NPC1L1 and ACAT2 protein; **b** Quantification of ABCG5 expressed as the ratio (as a percentage) of control; **c** Quantification of ABCG8 expressed as the ratio (as a percentage) of control; **d** Quantification of NPC1L1 expressed as the ratio (as a percentage) of control; **e** Quantification of ACAT2 expressed as the ratio (as a percentage) of control. Values shown are means ±SD, n = 4. *p < 0.05 and **p < 0.01 versus LD group

As shown in Fig. 5, pioglitazone rather than ezetimibe obviously increased the expression of ABCG5 and ABCG8 (**P < 0.01), and pronouncedly inhibited the LD-induced elevation of NPC1L1 and ACAT2 in the intestine (NPC1L1: **P < 0.01; ACAT2: *P < 0.05, **P < 0.01). These results suggest that pioglitazone would reduce cholesterol absorption in intestine.

### Pioglitazone changed PPARγ, LXRα and FXR in the liver or intestine

**Fig. 6:**
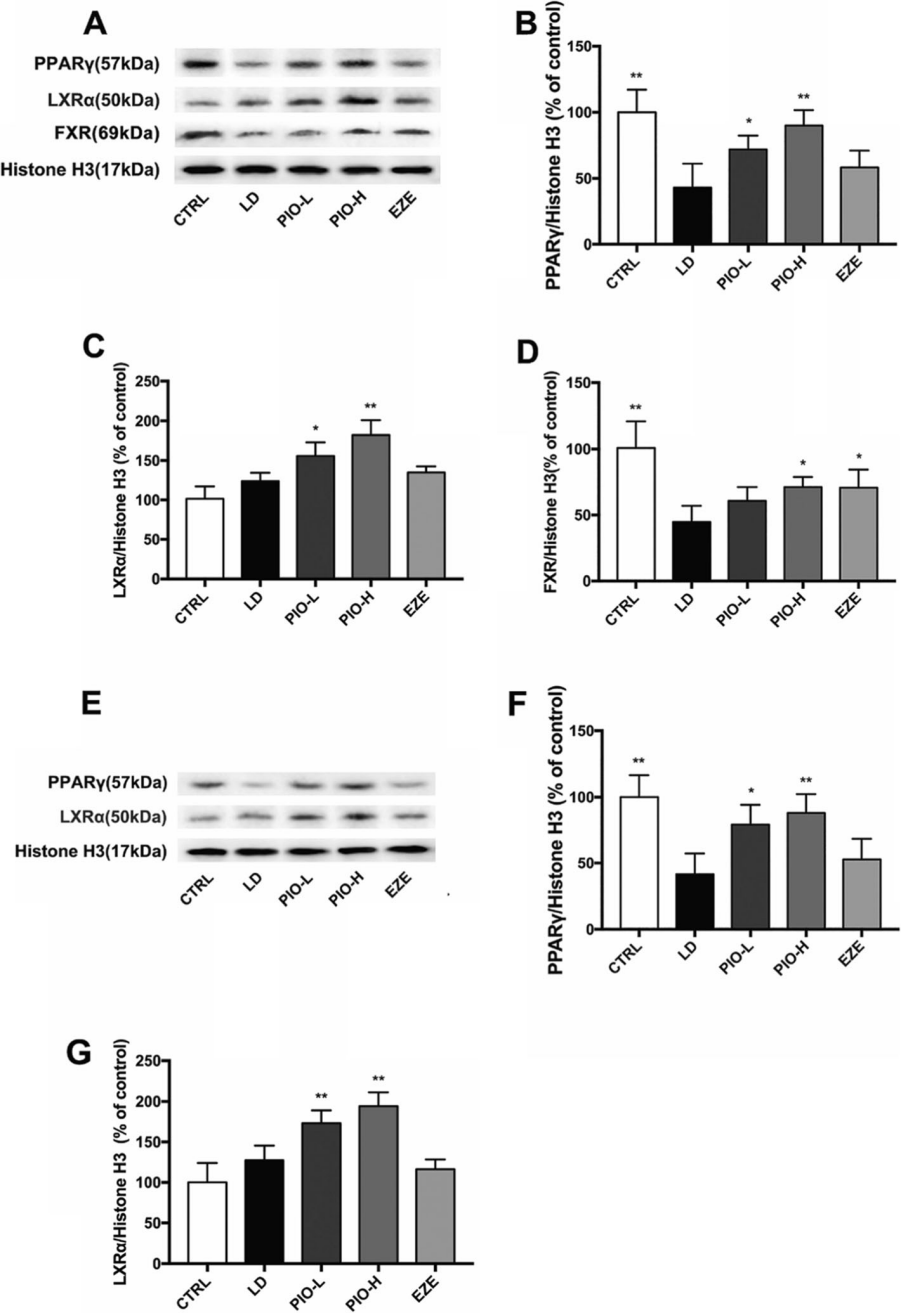
Pioglitazone changes PPARγ, LXRα and FXR in liver or intestine. **a** Western blot analysis of PPARγ, LXRα and FXR protein in liver; **b** Quantification of PPARγ expressed as the ratio (as a percentage) of control; **c** Quantification of LXRα expressed as the ratio (as a percentage) of control; **d** Quantification of FXR expressed as the ratio (as a percentage) of control; **e** Western blot analysis of PPARγ and LXRα protein in intestine; **f** Quantification of PPARγ expressed as the ratio (as a percentage) of control; **g** Quantification of LXRα expressed as the ratio (as a percentage) of control. Values shown are means ±SD, n = 4. *p < 0.05 and **p < 0.01 versus LD group

As shown in Fig.6, pioglitazone rather than ezetimibe obviously increased expression of PPARγ and LXRα in both liver and intestine (liver: PPARγ: *P < 0.05, **P < 0.01; LXRα: *P < 0.05, **P < 0.01; intestine: PPARγ: *P < 0.05, **P < 0.01; LXRα: **P < 0.01), while pioglitazone and ezetimibe obviously inhibited LD-induced decrease in expression of FXR in the liver (*P < 0.05), suggesting that LXRα and FXR might be involved in regulation of the expression of the genes related to cholesterol homeostasis.

## Discussion

The present study clearly indicated that pharmacological treatment with pioglitazone effectively prevented the formation of CGD induced by the LD in guinea pigs, accompanied by decreased CH, increased BA, and lower CSI in the bile as well as lower blood glucose, insulin, TG and CH levels. Pioglitazone also ameliorated hepatic and gallbladder tissue damage induced by the LD, which could also influence the formation of CGD. The further studies showed that pioglitazone treatment resulted in remarkable decreases in NPC1L1 and ACAT2, and increases in ABCG5/8 in the ileum as well as significant decreases in HMGCR and SREBP2, increases in CYP7A1, ABCG5/8, and BSEP in the liver. All of these effects are contributive to restoration of cholesterols homeostasis and prevention of the CGD.

The formation of CGD refers to three main pathophysiological mechanisms including the supersaturation of bile with cholesterol, gallbladder hypomotility and destabilization of bile by proteins or pathogens. Among these, cholesterol supersaturation of bile remains the most critical determinant of the CGD. During the process of the CGD, the homeostasis of cholesterol is highly disrupted, and events involve cholesterol intake, metabolism, and synthesis. It is known that cholesterol derived from the intestine provides the first major source for cholesterol pool and influences biliary cholesterol secretion [20], and that high absorption of cholesterol contributes to gallstone formation through this pathway [21]. Animal studies indicate that high cholesterol absorption efficiency and subsequent rapid biliary secretion of cholesterol promote cholesterol gallstone disease [21]. The LD-induced CH gallstone models are widely used to elucidate the mechanism of CGD pathogenesis [22, 23]. Herein, administration to guinea pigs for 8 successive weeks disrupted the homeostasis of cholesterol indicated by higher levels of CH in both the blood and the bile, which results mainly from the increase in cholesterol intake. Our results showed that the LD-fed guinea pigs had a 85.7% gallstone formation rate. The present data also further confirmed the preventive effects of pioglitazone on the CGD in the model of guinea pigs as shown in the mouse model [24]. As a key protein controlling intestinal cholesterol absorption, NPC1L1 was expressed by enterocytes, identified as a target of ezetimibe [25]. Ezetimibe, a selective NPC1L1 inhibitor, prevents gallstone formation in mice, indicates that NPC1L1 is a valid therapeutic target against the CGD [26]. Free cholesterol absorbed in the intestine through NPC1L1 forms a cholesterol ester catalyzed by ACAT2 and then enters the blood circulation through the lymphatic system, while the unesterified cholesterol is secreted into the intestinal lumen by ABCG5/8, which is the opposite to action of NPC1L1. In the present studies, we found that LD induced significant upregulation of both NPC1L1 and ACAT2 rather than ABCG5/G8 in the ileum, and pioglitazone, like ezetimibe, markedly inhibited expression of NPC1L1 and ACAT2. Importantly, pioglitazone instead of ezetimibe significantly increased intestinal ABCG5/G8 expression. These suggest that pioglitazone might more effective than ezetimibe in reduction of cholesterol intake in intestine.

The hepatocyte is the major site for peripheral uptake and synthesis of cholesterol, and excess cholesterol is directly secreted into bile or converted into bile salts [27]. Endogenous synthesis of cholesterol is regulated at the transcriptional level by the SREBP-2 pathway and is regulated at the post-transcriptional level by the rate-limiting enzyme HMGCR [28]. When the intracellular cholesterol level is high, SREBP-2 forms a complex structure with Scap (SREBP cleavage-activating protein) and Insig-1 (insulin-induced gene-1) through the C-terminal domain, thereby inhibiting SREBP-2 regulation of HMGCR transcription. Our data also showed that expression of HMGCR or SREBP-2 was decreased through feedback regulation in the guinea pigs fed with LD, and pioglitazone instead of ezetimibe make HMGCR and SREBP-2 further deceases in the liver. These are supported by the previous data that PPARγ activation caused reductions of the SREBP-2 and its target genes HMGCR, as well as the reduction of cholesterol synthesis in vitro [29, 30]. Synthesis of bile acids from cholesterol is the dominant pathway for eliminating excess cholesterol in the body. Bile acids may reduce the synthesis of itself through feedback inhibition of CYP7A1, when they are too much [31]. It is known that the expression of CYP7A1 can be regulated by several negative feedback loops, such as FXR-SHP-LRH-1 pathway [32]. It was found that there is a feedback repression of CYP7A1 when the guinea pigs fed with LD, and both of pioglitazone and ezetimibe reversed this inhibition in the present studies. The conversion of cholesterol into bile acids is regulated by LXRα-mediated stimulation of CYP7A1 transcription [33]. LXRα is the predominant receptor in the liver, and is directly regulated by PPARγ [34, 35]. Therefore, this might explain the up-regulation of CYP7A1 after PPARγ upregulation resulting from the treatment with pioglitazone. BSEP is the major hepatic BA canalicular efflux transporter. Both of pioglitazone and ezetimibe increased gallbladder biliary BA content, which is involved in the enhancement of BSEP-mediated BA transport.

ABCG5 and ABCG8 are target genes of LXRs [7, 36]. LXRα is directly regulated by PPARγ [34], and thus this might explain how LXRα cooperates with PPARγ to expedite cholesterol efflux in the intestine and liver. FXR plays a key role in the regulation of BA levels in enterohepatic circulation and has been shown important in preventing gallstone formation in susceptible mice. FXR deficient mice were found to be more susceptible to cholesterol gallstone formation than wild-type mice when fed with a lithogenic diet [37]. Moreover, treatment of C57L mice, which are susceptible to cholesterol gallstone formation, with a potent FXR agonist prevents gallstone formation [37]. FXR activation also reduced hepatic expression of sterol regulatory element binding protein 1c, resulting in reductions of triglyceride and cholesterol content in the liver and amelioration of hyperlipidemia [38]. There is a cross-talk between FXR and PPARγ, which is necessary for a proper response to PPARγ activation [39]. Thus, FXR also participate in the improvement of cholesterol homeostasis by pioglitazone.

## Conclusions

**Fig. 7:**
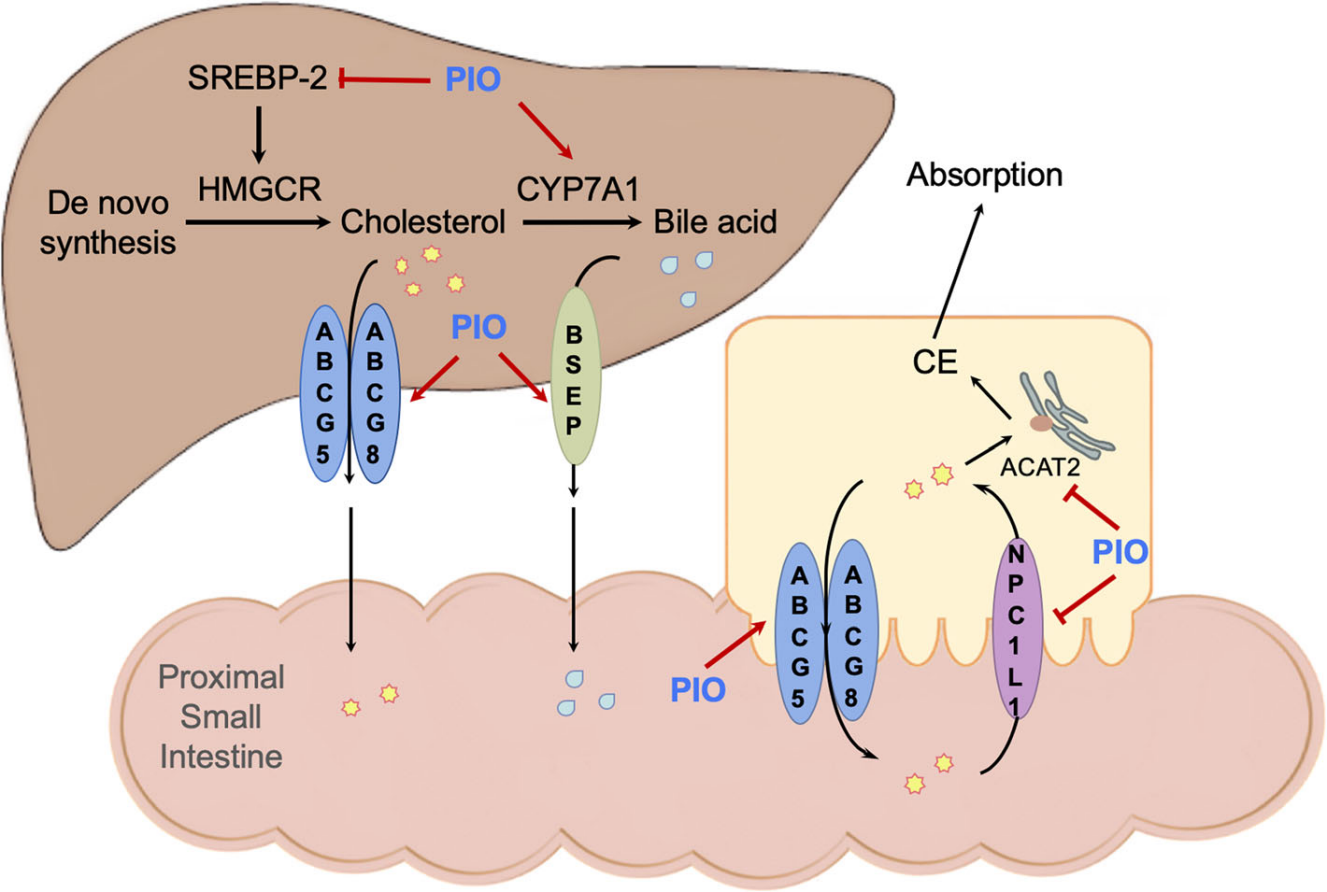
The illustration shows the mechanism of pioglitazone (PIO) on regulation of cholesterol homeostasis

In summary, as shown in Fig. 7, as a PPARγ agonist, pioglitazone prevented the LD-induced formation of the CGD, and restored cholesterol homeostasis. These effects are associated with regulating expression of HMGCR and SREBP2, CYP7A1, ABCG5/8, BSEP, NPC1L1 and ACAT2 in the liver or intestine as well as amelioration of hepatic and gallbladder tissue damage induced by the LD. PPARγ, LXRα and FXR participated in regulation of these genes associated with absorption, synthesis, transformation, and transportation of CH together or independently. We thus suggest that PPARγ agonist pioglitazone might have beneficial effects for the CGD.

## Data Availability

All data generated or analyzed during this study are included within the article.

## Abbreviations

ABCG5, ABCG8: Adenosine triphosphate-binding cassette (ABC) sterol transporters G5 and G8
ACAT2: Acetyl-Coenzyme A cholesterol acyltransferase
BA: Bile acid
BSEP: Bile salt export pump
CGD: Cholesterol gallstone disease
CH: Cholesterol
CSI: CH saturation index
CYP7A1: 7α-hydroxylase
EZE: Ezetimibe
HDL-C: High-density lipoprotein CH
HMGCR: Hydroxyl-methyl-glutaryl-CoA reductase
LD: Lithogenous diet
LDL-C: Low-density lipoprotein CH
LXRα: Liver X receptor α
NPC1L1: Niemann-Pick C1-like 1
PIO: Pioglitazone
PL: Phospholipid
PPARγ: Peroxisome proliferator-activated receptor-γ
SREBP2: Sterol regulatory element-binding proteins 2
TG: Triglyceride
TZD: Thiazoledinedione
